# Analysis of phenolic compounds in commercial dried grape pomace by high-performance liquid chromatography electrospray ionization mass spectrometry

**DOI:** 10.1002/fsn3.136

**Published:** 2014-06-15

**Authors:** Lina M Ramirez-Lopez, Christina A M DeWitt

**Affiliations:** 1 Department of Animal Science and the Robert M. Kerr Food & Agricultural Products Center, Oklahoma State UniversityStillwater, Oklahoma, 74078; 2 Department of Food Science and Technology, Seafood Research and Education Center, Oregon State UniversityAstoria, Oregon, 97103

**Keywords:** Commercial dried grape pomace, extraction, HPLC-ESI-MS, phenolic compounds

## Abstract

By-products obtained from winemaking processes still contain large amounts of phenolic compounds, especially phenolic acids, flavanols, flavonols, stilbenes, and flavonoids. Enzymatic hydrolysis was used for determination and characterization of phenolic acids, flavanols, flavonols, and stilbenes. Characterization of the flavonoids was achieved using acid hydrolysis with 0.1% hydrochloric acid. In addition, organic solvents as 50% methanol, 70% methanol, 50% acetone, 0.01% pectinase, and 100% petroleum ether were also evaluated. Reversed phase high-performance liquid chromatography (RP-HPLC) with diode array detector was used to identify phenolic compounds. Internal standard quantification was implemented using a five points of the UV-visible absorption data collected at the wavelength of maximum absorbance. A total of 16 phenolic compounds were determined. The content differed from 1.19 to 1124 mg kg^−1^. Outcomes from HPLC study showed that gallic acid, (+) catechin hydrate, and (−) epicatechin gallate were the major phenolic compounds presented in the sample. Malvidin and pelargonidin 3-*O*-glucoside were the major anthocyanins monoglucosides.

## Introduction

Cabernet grape has been considered one of the world's widely recognized red wine grape varieties. It is characterized by growing in every major wine production country with a several range of climates and ease of cultivation. The countries with the main production of Cabernet grape include: Canada, France, Spain, Italy, New Zealand, Sidney, Chile, Argentina, Bolivia, some States in USA such as California, East Washington (Pazourek et al. 2005). The cultivar has been characterized to have thick skins and the creepers are hard and resistant to deterioration and ice (Makris et al. 2007). Grape pomace (a by-product containing skin and seeds) is one of the most plentiful remains of the winemaking process. Grape remains (stems, skins, and seeds) are recognized as storage of important chemical compounds such as phenolic acids, flavonoids, stilbenes, organic acids, and sugars (Pinelo et al. 2005; Lafka et al. 2007). In the past few years in studying and quantifying the phenolic compounds of red fruits have increased. Grape phenolics include a wide range of compounds with antioxidant activity, classified as flavanols, flavonols, phenolic acids, stilbenes, and anthocyanins (Bowyer 2002). The concentration and composition of phenolics in red wine grapes vary with species, variety, season, and a wide range of environmental and management factors such as climate, soil conditions, canopy management, and crop load. The extraction of phenolic compounds is primarily influenced by their sample particle size, the extraction method, and storage time (Maier et al. 2008).

The objectives of this study included: (a) to identify and quantify the main phenolic compounds in commercial dried grape pomace using high-performance liquid chromatography (HPLC); (b) to compare the recovery of the main phenolic compounds in commercial dried grape pomace using conventional polar solvents: 50% methanol–water mixture, 70% methanol–water mixture, 50% acetone–water mixture, 100% petroleum ether, and 0.01% pectinase solutions.

## Experimental

### Chemicals

The following standards were purchased from Fluka (St Louis, MO): gallic acid, ferulic acid, caffeic acid, *p*-coumaric, (+) catechin hydrate, quercetin, (−) epicatechin gallate, isorhamnetin, myricetin, *trans*-resveratrol, 7-ethoxycoumarin, and *β*-glucosidase. The 3-*O*-glucosides of delphinidin, cyanidin, petunidin, pelargonidin, peonidin, and malvidin were obtained from Polyphenols Laboratories AS (Sandnes, Norway). Sep-Pak C_18_ cartridges (1 g, 6 mL) were obtained from Waters Corporation (WAT051910; Waters Corp., Milford, MA). HPLC grade methanol, acetonitrile, acetone, petroleum ether, and phosphoric acid were purchased from Fisher Scientific (Fair Lawn, NJ). Water was from Milli-Q purification system Millipore (Millipore, Bedford, MA). Ascorbic acid, ethyl acetate, and *β*-glucosidase type HP-2 from *Helix pomatia* were purchased from Sigma Aldrich (St. Louis, MO).

### Samples

Commercial dried grape pomace was obtained from a wine industry localized in Canada. Sample was vacuum packed upon arrival.

### Grape sample extractions

#### Extraction procedure I (flavonols, flavanols, phenolic acids, and stilbenes)

The extraction procedure I for flavonols, flavanols, phenolic acids, and stilbenes (FFPAS) was modified from the previous work of Torres et al. (2005). Briefly, 0.5 g of commercial dried sample was weighed (A-160, Analytical balance, Denver Instruments Co, Denver, CO) and transferred to a 30-mL brown bottle (glass amber with teflon face lined cap, Fisherbrand, ThermoFisher Scientific Inc., Minneapolis, MN). The internal standard extraction was done by placing 25 *μ*L of 25 ppm 7-ethoxycoumarin (internal standard) in 4 mL of 50% v/v methanol–water mixture. The final solution was placed in an ice bath, stirred for 1 h and subsequently centrifuged (Clinical 50-82013-800 centrifuge VWR International, Chicago, IL) at 3000*g* for 20 min. The centrifuged solution was decanted using Whatman filter paper (# 41) and placed in a 10-mL volumetric flask. Sample was then re-extracted under the same conditions and the combined filtrates were brought to volume (10 mL of 50% methanol–water mixture). A final sample of 2 mL was placed into a brown vial (3 mL capacity, glass amber with teflon face lined cap, Fisherbrand, ThermoFisher Scientific Inc.), where 100 *μ*L of ascorbic acid, 50 *μ*L of *β*-glucosidase, and 110 *μ*L of 0.78 mol/L acetate buffer (pH 4.8) were added. Sample was vortexed, incubated at 37°C for 17 h (overnight), and then it was centrifuged at 4000*g* for 25 min. The final sample was analyzed by reversed phase high-performance liquid chromatography (RP-HPLC; Thimothe et al. 2007).

#### Extraction procedure II (anthocyanin monoglucosides)

The extraction procedure II was adapted from Kammerer et al. (2004) with some modifications. Briefly, 5 g of sample were combined with 200 *μ*L of the internal standard 25 ppm (7-ethoxycoumarin) and 100 mL of methanol/0.1% HCl (v/v) in a bottle and under stirring were mixed for 1 h. Subsequently, the solution was flushed with nitrogen in order to prevent oxidation during extraction at room temperature. After extractions, the final extract was centrifuged at 4000*g* for 10 min and the sample was re-extracted with 100 mL of the organic solvent under the same conditions for 15 min. A final sample of the combined supernatants (5 mL) was evaporated to dryness in a nitrogen water bath (Zymark TurboVap, Zymark Center, Hopkinton, MA) at 30°C to remove the organic solvent. Therefore, the precipitate was dissolved with 2 mL of acidified water (acetic acid, pH 3.0) and analyzed by RP-HPLC (Thimothe et al. 2007).

#### Extraction with organic solvents (phenolic compounds)

Time, solvent-to-solid ratio, and temperature were based on literature data (Ju and Howard 2003) and prior extraction experiments of the research group (Vassan 2009). Variables during the extraction tests were ratio of 40-mL solvent per 20 g of extraction material and extraction time 1 h. The polar solvents were eliminated by evaporation using nitrogen as a carrier at 35°C. The conventional extraction solvents were 50% acetone–water, 70% methanol–water, petroleum ether, and 0.01% pectinase–water mixture. Samples were not hydrolyzed.

Extractions using the organic solvents (40 mL) were conducted by weighing 20 g of commercial pomace powder into 125-mL Erlenmeyer flasks. In addition, the flasks were placed in a shaker (Classic C76, New Brunswick Scientific, Edison, NJ) at 18°C and 250 rpm for 1 h. After agitating, samples were filtered under vacuum using a Buchner funnel with 5.5-cm diameter (55 mm #1, Whatman Inc. Ltd., Mainstone, England). The petroleum ether extracts were allowed to evaporate and were resuspended in 100% acetone. The final filtrates using the rest of the solvents were transferred to 100-mL volumetric flask and brought up to volume. A sample of 10 mL was then evaporated to dryness in a water bath using nitrogen at 30°C. After evaporation, the precipitate was subsequently dissolved in 7 mL of Milli-Q water and applied to solid phase extraction cartridges (SPE, WAT051910, Waters Corp., Milford, MA). Samples of 5 mL were applied to the SPF cartridges, which were previously activated with methanol, rinsed with deionized water, and 0.1% HCl (v/v). The collected samples eluted from the cartridges were filtered through 0.45-*μ*m nylon filters (Fisherbrand, PTFE, Fisher Scientific, Denver, CO) and employed for RP-HPLC analysis.

### Chromatography analysis

The RP-HPLC procedure used in this study was modified from Thimothe et al. (2007). The method was developed in order to identify and quantify 17 phenolic compounds. The main FFPAS, standards were received as single compounds. The main flavonoids monoglucosides (anthocyanin monoglucosides [AM]) standards were received as a blend. A stock solution containing all the individual FFPAS and AM blend was prepared at 100 ppm using an internal standard (7-ethoxycoumarin) at 25 ppm. The standard curve was done by serially diluting (1:1) to a final concentration of 0.78 ppm. Characterization of phenolic compounds was achieved by using a reversed phase chromatography system (Alliance Waters 2690, Waters, Ireland) with a photodiode array detector (PDA, Waters 2996) and Empower 2 software (waters). A gradient elution system was used for separation of individual compounds on a Sun Fire™ C18 column (5-*μ*m particle size, 4.6 × 250 mm i.d.) including a guard column (5-*μ*m particle size, 4.6 × 30 mm) at 25°C. The flow rate was set to 1.0 mL/min. The mobile phases A and B were employed as follows: mobile phase A contained 0.1% H_3_PO_4_ in MilliQ water and mobile phase B contained 0.1% H_3_PO_4_ in acetonitrile (HPLC grade). Data acquisition was applied for 45 min with a total run of 65 min. Gradient elution was as follows: 92% A/8% B, at 0 min; 85% A/15% B at 5 min; 40% A/60% B at 45 min; 40% A/60% B at 55 min; and back to initial conditions 92% A/8% B at 60 min. The PDA was set at 210–600 nm and chromatograms were extracted at 280, 320, 370 nm for phenolic acids, stilbenes, and flavonoids, and 520 nm for AM.

### Electron spray ionization mass spectrometry

The mass spectrometry (MS) was a linear triple quad (LTQ) ion trap mass spectrometer (Thermo Scientific) equipped with an electrospray ionization (ESI) source. For detection of FFPAS, the negative ion mode (*m/z* − H^−^) was used. In addition, for detection of AM, positive ion mode (*m/z* M + H^+^) was used. Mass scan range was from 100 to 700 *m/z*. The fragmentation of MS/MS was performed to determine the charge of state of the phenolic compounds. The identification of the compounds was acquired by comparing their molecular ions (*m/z*) obtained by ESI-MS/MS with the standards. Nitrogen was used as a gas carrier at flow rates of 11 L/min and pressure was sat 70 psi. Helium was used as collision gas for the high-collision dissociation (HCD) at pressure of 3.0 × 10^−6^. Mass spectrometry study was used only for extraction procedures I and II.

### Statistical analysis

Data were analyzed using analysis of variance to determine differences among extraction of organic solvent means using the function PROC GLM of Statistical Analysis version 9.3 (SAS Institute, Cary, NC, 2003). Multiple comparison among the five organic solvents (50% methanol, 70% methanol, 50% acetone, 0.01% pectinase, and petroleum ether) were analyzed each with two analytical replicates. Means were separated by Tukey's test method (*P* < 0.05). All experiments were conducted in triplicate.

## Results and Discussion

### Chromatography analysis

Several factors such as retention time, maximum absorbance, mobile phases, and concentration were studied to develop a method capable of resolving a large number of the phenolic compounds that are present in grape pomace.

### Extraction procedure I (flavanols, flavonols, phenolic acids, and stilbenes)

In order to evaluate the efficacy using organic solvents of the commercial grape pomace powder, data were collected from extraction procedures I and II. These extractions of phenolic compounds from grape pomace have been previously reported (Kammerer et al. 2004; Thimothe et al. 2007). The extractions typically include the utilization of enzymatic hydrolysis to simplify chromatographic data. The enzyme *β*-glucosidase from *H. pomatia* type HP-2 is used to cleave the sugar moiety of phenolic glycosides (Yáñez et al. 2007). In addition, it was reported that *β*-gluconidase contained arylsulfatase activity and can also effectively deconjugate flavonoid glucosides in red fruits (Araújo et al. 2007).

**Figure 1:**
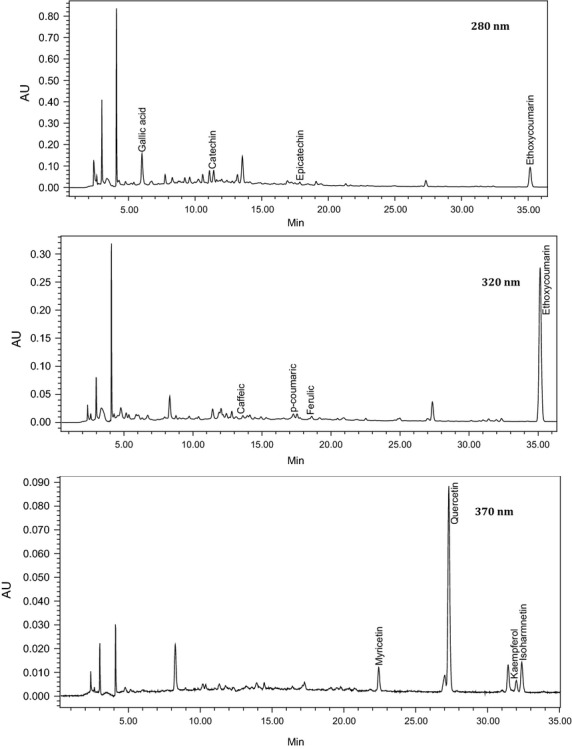
HPLC-PDA chromatogram (280, 320, and 370 nm) of the main phenolic compounds in commercial dried grape pomace using extraction procedure I.

The levels of individual and total FFPAS (flavanols, flavonols, phenolic acids, and stilbenes) and AM (anthocyanin monoglucosides) measured in pomace powder are displayed in Table 1. The FFPAS concentration measured in pomace was 1124 mg/kg dry matter using 50% methanol, which is in agreement with the results found by Rockenbanch et al. (2011), who reported total phenolic composition of 1065 mg/100 g catechin equivalent dry matter in Cabernet Sauvignon grape pomace extract. Figure 1 shows a typical separation of phenolic acids, stilbenes, and flavonols using extraction procedure I at 280, 320, and 370 nm.

**Table 1:** Content of phenolic compounds identified in commercial dried pomace (mg/kg ± RSD) using extraction procedures I and II.

Analyte	50% Acetone	70% Methanol	0.01% Pectinase	Petroleum ether	Extraction procedures I and II
FFPAS					
(−) Epicatechin gallate	46.00 ± 0.62	13.64 ± 0.19	2.40 ± 1.40	17.63 ± 0.41	52.39 ± 0.11
(+) Catechin hydrate	29.06 ± 0.55	16.93 ± 0.07	18.35 ± 0.63	1.65 ± 0.38	423.82 ± 0.19
Caffeic acid	0.71 ± 0.30	0.33 ± 0.14	0.34 ± 0.25	0.10 ± 0.04	10.61 ± 0.24
Ferulic acid	2.73 ± 1.11	1.22 ± 0.34	1.75 ± 0.85	9.25 ± 0.26	19.14 ± 0.02
Gallic acid	12.41 ± 0.25	15.36 ± 0.34	47.72 ± 0.40	<0.1	893.00 ± 0.04
Isorhamnetin	8.37 ± 1.05	11.73 ± 0.51	<0.1	1.05 ± 0.16	13.77 ± 0.47
Kaempferol	2.02 ± 0.90	2.37 ± 0.57	1.05 ± 0.01	1.38 ± 0.16	35.59 ± 0.17
Myricetin	1.98 ± 0.98	0.86 ± 0.65	0.41 ± 0.03	0.73 ± 0.11	19.52 ± 0.20
*p*-coumaric acid	8.22 ± 0.86	3.54 ± 0.56	0.91 ± 0.47	3.01 ± 0.38	27.06 ± 0.37
Quercetin	25.88 ± 0.94	13.90 ± 0.73	0.97 ± 0.42	11.05 ± 0.26	52.99 ± 0.58
*Trans*-resveratrol	0.86 ± 0.16	0.28 ± 0.19	<0.1	<0.1	<0.1
Totals	138.78 ± 14.85^b^	80.16 ± 6.90^b^	73.9 ± 15.88^b^	45.85 ± 6.13^b^	1124.07 ± 288.46^a^
AM					
Cy3G	1.13 ± 0.01	4.35 ± 0.77	0.84 ± 0.01	0.20 ± 0.01	3.17 ± 0.54
Dp3G	0.68 ± 0.02	2.41 ± 0.78	0.16 ± 0.08	0.06 ± 0.03	8.38 ± 0.59
Mv3G	2.19 ± 0.59	13.31 ± 1.15	0.20 ± 0.13	0.32 ± 0.01	4.85 ± 0.70
Pe3G	2.10 ± 0.38	<0.1	<0.1	<0.1	0.77 ± 0.38
Pg3G	2.32 ± 0.55	14.01 ± 0.61	0.48 ± 0.16	0.26 ± 0.05	15.66 ± 0.68
Pt3G	1.17 ± 0.82	2.57 ± 0.65	<0.1	0.35 ± 0.03	0.27 ± 0.01
Totals	9.59 ± 0.69^b^	36.65 ± 5.83^a^	1.68 ± 0.31^b^	1.19 ± 0.11^b^	33.10 ± 5.78^a^

Data are the mean for three replications ± RSD. Results are reported on a dry matter basis. <0.1 Lower than the detection limit 0.1 mg/kg. Means with similar letter (^a,b^) are not significantly different (Tukey, *P* > 0.05). FFPAS, flavonols, flavanols, phenolic acids, and stilbenes; AM, anthocyanins monoglucosides.

### Extraction procedure II (AM)

**Figure 2:**
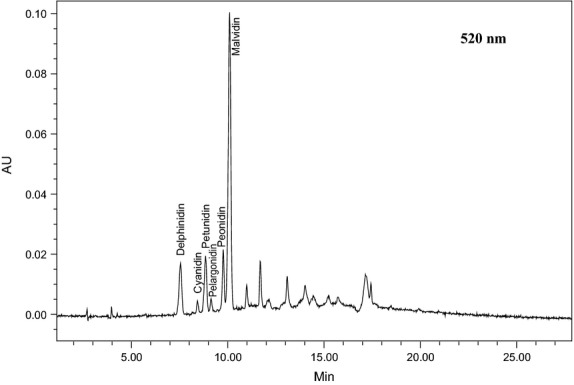
HPLC-PDA chromatogram (520 nm) of the anthocyanin monoglucosides in commercial dried grape pomace.

Anthocyanins are glucosides of anthocyanidins. Previous studies have categorized more than 15 anthocyanidins glycones (Harborne and Williams 2000). In this study, only AM (six of them) were identified and corresponded to cyanidin 3-*O*-glucoside (Cy3G), delphinidin 3-*O*-glucoside (Dp3G), malvidin 3-*O*-glucoside (Mv3G), pelargonidin 3-*O*-glucoside (Pg3G), peonidin 3-*O*-glucoside (Pe3G), and petunidin 3-*O*-glucoside (Pt3G) present in red fruits. Weak acids in combination with organic solvents can cause the hydrolysis of glucoside groups attached to flavonoids, thus, increasing their migration into the solvent (Gao and Mazza 1995). As can be observed, the recovery of AM was lower than for FFPAS. Hogan et al. (2009) found that total anthocyanins were lower than total phenolics in Cabernet Franc grapes with values of 0.64 C3GE (cyanidin 3-glucoside equivalent) mg/g and 28.1 GAE (gallic acid equivalent) mg/g, respectively. Figure 2 shows a typical separation of anthocyanins monoglucosides using extraction procedure II at 520 nm.

### Individual profile and tentative phenolic compounds by ESI-MS

Table 2 shows the mass data of FFPAS and AM compounds extracted from the commercial dried grape pomace using extraction procedures (I and II). Additionally, the presence of other main phenolic compounds was determined using extraction procedure I. These compounds included hydroxybenzoic acids (quinic acid, syringic acid, vanillic acid, *p*-hydroxybenzoyl glucoside, 3,4 dihydroxyphenylacetic acid); hydroxycinnamic acids (caffeoylshikimic acid, caftaric acid, cinnamic acid, fertaric acid); flavanones (naringenin). These findings are similar to the nonflavonoid content found by previous studies (Perestrelo et al. 2012).

**Table 2:** Identification of phenolic compounds in commercial dried grape pomace (extraction procedures I and II) by ESI-MS.

Tentative identification	MS (*m/z)*	MS/MS ions	MW
FFPAS [M]^−^			
Epicatechin gallate^1^	441	331/289/169	442
Catechin hydrate^1^	302		303
Caffeic acid^1^	179	135	180
Ferulic acid^1^	193	134	194
Gallic acid^1^	169	125	170
Isorhamnetin^1^	315	315	316
Kaempferol^1^	285	257	286
Myricetin^1^	317	317	318
*p*-coumaric acid^1^	163	119	164
Quercetin^1^	301	151/179	302
*p-*hydrobenzoic acid	137	107/93/79/53	138
Protocatechuic acid	153	109	154
Coniferyl aldehyde	177	149/133/105/89/77	178
Vanillic acid	167	123/107	168
3,4 Dihydroxyphenylacetic acid	167	125/123/107/99/89	168
Syringic acid	197	153/182	198
Cinnamic acid	147		148
Quinic acid	191	173/127/111/85	192
Catechin	289	245/205/179	290
Epicatechin	289	245/169	290
Phloretin	273	163	274
Caftaric acid	311	179/135	312
Caffeoylshikimic acid	335	179/161/135	336
*Bis*-HHDP-hexose	391	481/301/257	392
Quercetin 3-*O*-glucoside	493	301	494
Naringenin 7-*O*-glucoside	433	271	434
AM [M]^+^			
Cyanidin 3-*O*-glucoside^1^	449	287	448
Delphinidin 3-*O*-glucoside^1^	465	303	464
Malvidin 3-*O*-glucoside^1^	493	331	492
Pelargonidin 3-*O*-glucoside^1^	433	271	432
Petunidin 3-*O*-glucoside^1^	479	317	478
Peonidin 3-*O*-glucoside^1^	462	301	461
Delphinidin 3,5 diglucoside	627	465/303	626
Cyanidin 3 (acetylglucoside)	491	287	490
Delphinidin3-*O*-*β*-glucopyranoside	465		464
Malvidin 3-gentiobiside	665	331	664
New pigment B	677		676
Delphinidin 3-*O*-*p-*coumaryl glucoside	611	303	610
Petunidin 3-*O*-*p*-coumaryl glucoside	625	317	624
Malvidin 3-*O*-*p*-coumaryl glucoside	639	331	638
Peonidin-malonylglucoside	548	463/301	

FFPAS [M]^−^: Negative-ion mode for flavanols, flavonols, phenolic acids, and stilbenes. AM [M]^+^: Positive-ion mode for anthocyanin monoglucosides.

1 Identified using the corresponding authentic standards.

For identification of AM not only monoglucosides were detected in commercial dried grape pomace from Cabarnet grape but also additional flavonoid compounds were identified using extraction procedure II (Table 2). These findings were in agreement with previous studies conducted on Cabernet pomace. Ruberto et al. (2007) reported that the presence of major anthocyanin diglucosides, acylated anthocyanins, and coumaroylglucoside anthocyanins are characteristic of the methanol-acidified HCl in Cabernet pomace.

### Organic solvent extracts

Extraction of phenolic compounds from commercial dried pomace using acetone–water, methanol–water, and water has been previously reported (Ju and Howard 2003; Ryan and Revilla 2003; Lapornika et al. 2005). The success of organic solvent extractions in recovering FFPAS and AM from pomace was measured by comparing results to the previously described extraction procedures I and II. Significant differences were found among the mean of organic solvents used for extraction of phenolic compounds in commercial dried pomace (*P* < 0.05) (Table 1). The concentration of AM using 70% methanol showed the highest recovery 36 mg/kg dry matter. However, this solvent was not the most efficient recovering FFPAS. The highest concentration of FFPAS among the solvents was obtained using 50% methanol with recoveries of 1124 mg/kg dry matter.

### Evaluation of different extraction procedures of phenolic compounds

Both extraction procedures I and II for extracting phenolic compounds of commercial grape pomace achieved strongly in the recovery of phenolic compounds. The extraction procedures I and II were higher in recovering phenolic compounds than the conventional extraction solvents. The recovery of FFPAS and AM using 50% methanol and 70% methanol were the most effective (*P* < 0.05). The FFPAS values of extracts when using 50% methanol solvent were the highest among the other solvents (1124 mg/kg). However, the AM values of extracts when using 70% methanol was the highest among the other solvents (36 mg/kg). In previous studies researchers reported that red grape pomace not only has a high content of AM but also appears to have a higher content of unknown compounds (Thimothe et al. 2007). Comparable observations were made in the current study using the commercial grape pomace. These unknown peaks were also detected in this commercial dried grape pomace (Fig. 2) and can be related to acylated anthocyanins, which are present in abundance in grapes (Hong and Wrolstad, 1990).

## Conclusions

In the present study, characterization of the main phenolic acids, flavonoids, flavonols, stilbenes, and AM in commercial dried grape pomace were established by chromatographic techniques. The results confirm that commercial dried grape can be used to improve the value of the by-products from wine industry. In addition, these by-products could be for different applications such as ingredients, natural antioxidants, and color additives in the food industry. In addition, the present research provides useful information for selecting extraction methods different to the conventional organic solvent extractions as alternative of recoveries of phenolic compounds.
